# Succession in a Tropical Dry Forest: A Test of the Chronosequence and Inference of Community Assembly Dynamics

**DOI:** 10.1002/ece3.73895

**Published:** 2026-06-23

**Authors:** Mary E. Carrington, Michael S. Ross, Suresh C. Subedi

**Affiliations:** ^1^ Governors State University, College of Graduate Studies University Park Illinois USA; ^2^ Florida International University, SERC Miami Florida USA; ^3^ Department of Biology Norfolk State University Norfolk Virginia USA

## Abstract

To better understand successional changes and drivers of changes in forests, we must (1) test assumptions of successional studies and (2) quantify patterns in species and functional traits in stands varying in successional age. Successional changes are commonly approximated by quantifying changes along chronosequences, or space‐for‐time substitutions. These assumed successional patterns, however, are rarely tested through longitudinal studies of individual sites. To identify drivers of successional trajectories and to address questions of deterministic vs. stochastic community assembly, species composition data must be supplemented with data such as species functional traits. We examined patterns in species composition and functional traits along a chronosequence of tropical dry forest (TDF) sites in North Key Largo, Florida in 1993. To test patterns identified along the chronosequence, we quantified longitudinal change in species and functional traits by remeasuring the sites in 2013. We tested the following predictions: (1) dynamic changes within sites in species composition and functional trait composition will corroborate a pattern indicated by the chronosequence; (2) a growth‐survival demographic tradeoff will be consistent with increasing dominance of evergreen species over the course of succession; and (3) forest succession will demonstrate convergence in both composition and function. As predicted, dynamic changes in species composition and functional traits within sites corroborated patterns quantified over the chronosequence, and species functional groups showed a growth‐survival demographic tradeoff between deciduous species early in succession and evergreen species late in succession. Surprisingly, however, although species composition converged late in succession, functional traits showed greatest divergence late in succession. We suggest that this divergence is due to functionally disparate evergreen and leaf exchanger species coexisting with high abundances for the first time late in succession, and to interspecific competition in an environment of limited resources late in succession. *Synthesis.* This is the first study in TDF to corroborate compositional and functional changes measured along a chronosequence with results of longitudinal measurements within sites. Additionally, this TDF ecosystem shows a rare successional pattern of convergence in species composition but divergence in functional traits, likely due to the coexistence of different functional groups and interspecific competition late in succession.

## Graphical abstract

This successional study in Florida Keys tropical dry forest (TDF) is the first to corroborate compositional and functional changes measured along a chronosequence with results of longitudinal measurements within sites. Additionally, this TDF ecosystem showed a rare successional pattern of convergence in species composition but divergence in functional traits, likely due to the coexistence of different functional groups and interspecific competition late in succession.

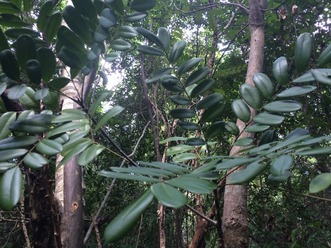

## 1 Introduction

Studies of succession—defined broadly as the temporal change in species composition or structure of an area's plant cover (Pickett and Cadenasso 2005)—often use major disturbances to mark the starting point of the process and infer dynamics from patterns revealed in a space‐for‐time substitution, or chronosequence. A chronosequence consists of a one‐time sample of a spatially segregated series of sites purported to differ only in time since disturbance. Inferences from chronosequences are bound to be imperfect, as they depend on assumptions that are rarely entirely true: that the initial physical and biological legacies are uniform among sites, and that species availability and environmental conditions are invariant through time (Johnson and Miyanishi 2008). Nevertheless, the sequence of species replacement indicated through a well‐designed forest chronosequence represents a first approximation of how the individual stands that comprise the sample, or other nearby forests, change over time after catastrophic disturbance. As a working hypothesis, it may be tested and later amended by tracking vegetation dynamics over time in the plots that formed the chronosequence, in effect adding a third dimension to the analysis (Foster and Tilman 2000; Chazdon et al. 2007). By combining these methods, questions of determinacy, directionality, and convergence in composition within the successional process can be addressed.

Succession may lead to gradual changes in environmental conditions in communities, such as those associated with canopy and soil developmental processes. In some ecosystems, however, these slow changes are accompanied by environmental disturbances that appear and dissipate rapidly but leave an enduring mark on the community. In subtropical ecosystems, tropical storms including hurricanes may cause such disturbances (Ross et al. 2001; Flynn et al. 2010). In south Florida in particular, colonization of hurricane‐caused canopy openings by invasive exotic species (primarily vines) may alter successional trajectories (Horvitz et al. 1995, 1998; Lynch et al. 2009).

If environmental processes operating at disparate scales (e.g., slowly changing environmental conditions over the course of succession, rapid changes caused by disturbance) combine to influence plant species composition and function, then interpretation of a successional chronosequence in isolation is likely to be unclear. By corroborating chronosequence data with direct observations of temporal change at individual sites with known histories and supplementing these changes in species composition with data on functional trait dynamics, it may be possible to better understand the multidimensional nature of community response (Kahmen and Poschlod 2004; Rolo et al. 2016). Although temporal changes within sites corroborated chronosequence changes in studies in several ecosystems (e.g., Chapin et al. 1994; Foster and Tilman 2000; Řehounková et al. 2024), including one tropical dry forest (TDF) study (Lebrija‐Trejos et al. 2010), another TDF study found that site age within a chronosequence was a poor predictor of temporal changes within sites early in succession (Mora et al. 2015). No study in TDFs has compared changes in functional traits across a chronosequence with changes within sites over time.

Generally, to identify environmental dynamics accompanying successional trajectories, Supporting Information beyond species compositional data is needed. Changes in the predominance of certain functional traits in sites over time may indicate the importance of biotic or abiotic factors during succession. In some forest types, limitation in resources (e.g., light, nutrients, water) increases over the course of succession, causing species that best tolerate these limitations, and their associated functional traits, to become more prominent over time (Uhl and Jordan 1984; Lohbeck et al. 2013, 2014; Zhang et al. 2015). In others, for example, infertile sites in which early‐to‐intermediate stages of secondary succession are characterized by accumulating stocks of organic matter and increasing nutrient availability, traits that result in fast growth rather than conservative resource use emerge later in succession (Berendse 1998).

Some TDFs appear to fit the latter model better, with canopy development leading to cooler conditions and greater soil moisture availability (Lohbeck et al. 2013; Pineda‐Garcıa et al. 2013). A recent successional study in TDFs in the Florida Keys, however, showed the opposite trend, with species traits early in succession typical of fast growth, while species traits later in succession were associated with conservative resource use. This trend in functional traits, along with overdispersion of traits among species in late successional stands, suggested that competition for limited resources was the predominant mechanism influencing community assembly late in succession (Subedi, Ross, et al. 2019). Moreover, this successional trend is consistent with results from a recent study of neotropical forests demonstrating a growth‐survival demographic trade‐off, with fast‐growing species abundant early in succession and slower‐growing species with high survival rates abundant late in succession (Ruger et al. 2023). Interestingly, however, the same study additionally identified a stature‐recruitment trade‐off between species with fast growth and high survival but low recruitment (long‐lived pioneers) and species with slow growth and low survival but high recruitment and identified long‐lived pioneers as a prominent component of canopies in mid‐ to late‐successional forests.

In Florida Keys TDFs, a growth‐survival demographic tradeoff during succession may be closely related to a leaf economics spectrum among tree species. Previous work has shown that species with deciduous leaves and evergreen leaves dominate early and late in succession, respectively (Ross et al. 2001), due to differing functional traits conferring evolutionary advantages during different stages of succession (Givnish 2002; Wright et al. 2004; Matsuo et al. 2024). Leaf‐exchangers, a third potential group in the leaf economics spectrum, shed their leaves just as often as deciduous species (once per year), but immediately replace their leaves and do not have a leafless period. Evolutionary advantages and behavior during succession for this group are less well understood (Givnish 2002).

Comparing functional beta diversity to species‐based beta diversity estimates over the course of succession can distinguish deterministic from stochastic community assembly mechanisms (Swenson et al. 2011, 2012; Yang et al. 2015; Buzzard et al. 2016; Muscarella et al. 2016). Whereas stochastic community assembly should result in functional beta diversity similar to species beta diversity, deterministic processes could result in two patterns of functional beta diversity relative to species beta diversity. Environmental filtering, or relatively constant local abiotic conditions late in succession, should result in lower functional beta diversity compared to species beta diversity. Alternatively, if the local environment is heterogeneous or changes rapidly late in succession (e.g., resulting from canopy gaps or short‐lived, acute disturbances), or if niche‐based community assembly processes result from interspecific competition, functional beta diversity should be high relative to species beta diversity (Swenson et al. 2012).

Previous studies of TDFs have documented changes in both species composition and functional traits over the course of succession (Lebrija‐Trejos et al. 2010; Lohbeck et al. 2013; Buzzard et al. 2016; Subedi, Ross, et al. 2019). In some of these forests, species composition showed convergence (Lebrija‐Trejos et al. 2010) or functional traits were not overdispersed (Buzzard et al. 2016), suggesting deterministic community assembly influenced by ecological filtering (Keddy 1992; Pillar et al. 2009; Mayfield and Levine 2010; Kraft et al. 2015). In a study conducted in Florida Keys TDFs, however, overdispersed functional traits late in succession indicated an influence of niche partitioning mechanisms on community assembly (Subedi, Ross, et al. 2019; see also Chesson 2000). Although studies in TDFs generally have not compared the magnitude of change during succession between species composition and functional traits, results of one study suggested that change in functional traits over the course of succession was greater in a dry tropical forest than in a wet tropical forest (Lohbeck et al. 2013).

**FIGURE 1:**
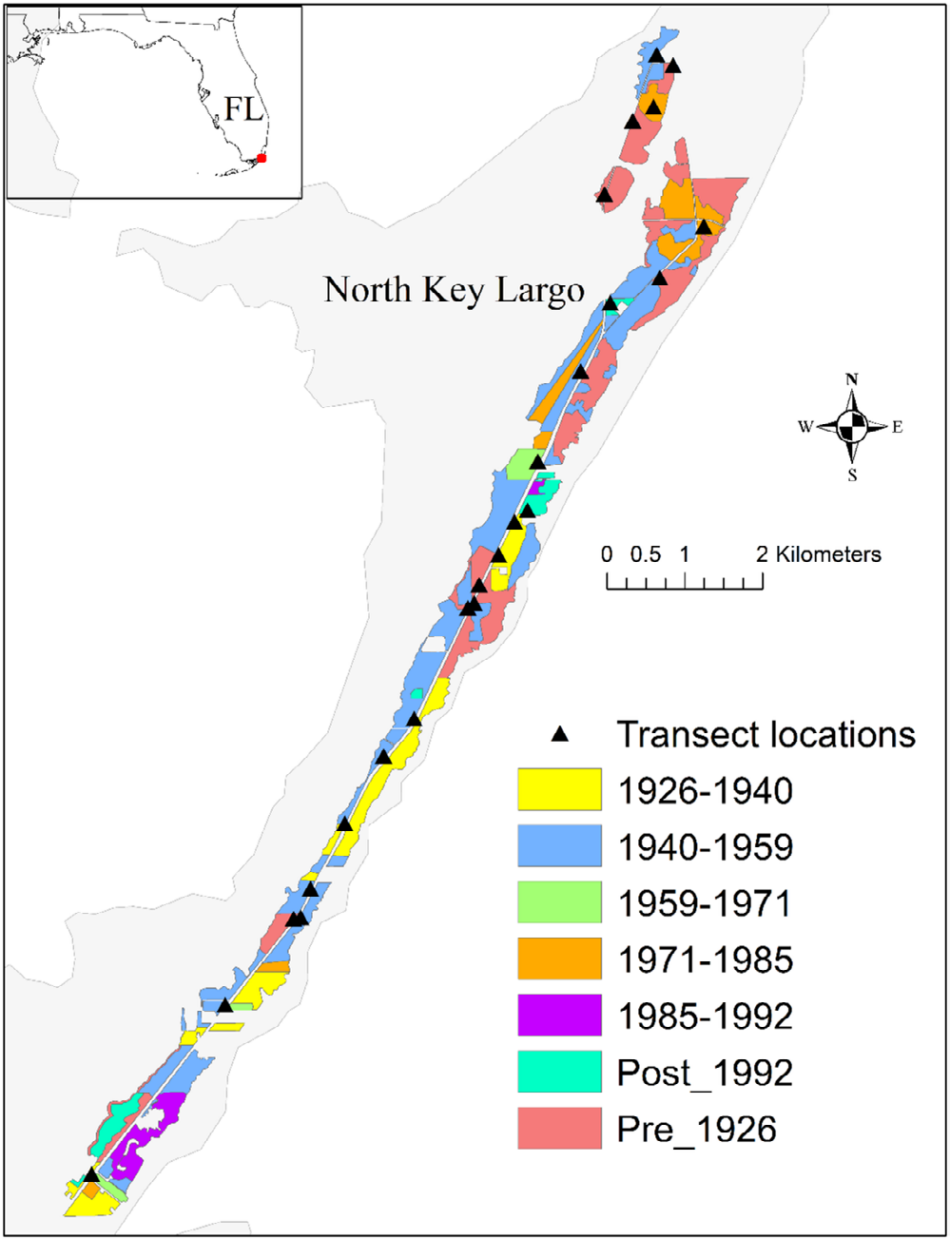
Study area with transect locations. Color codes indicate site disturbance year (i.e., start of secondary succession). Modified from Diamond and Ross (2020).

In the study described below, we examined the successional process in a TDF in the Florida Keys (Figure 1) over a chronosequence of sites, and by resampling each site in the chronosequence to obtain longitudinal data on species composition. We also collected data on site environmental conditions and tree species functional traits. Our study tested the following three predictions: (1) over a decadal‐scale period following disturbance, dynamic pathways of individual sites in species composition and species abundance‐weighted functional trait composition will follow a successional gradient indicated by the chronosequence; (2) a growth‐survival demographic tradeoff will be linked with species‐abundance‐weighted leaf trait changes consistent with increasing dominance of evergreen species over the course of succession, and (3) forest succession will entail a convergence in both composition and function (i.e., a decrease in across‐stand variation in the oldest forests).

## 2 Materials and Methods

### 2.1 Study Area

Warmed by the passing Gulfstream, the climate of Key Largo FL (25°14′ N, −80°20′ W) places it within Holdridge's (1967) Dry Tropical Forest Life Zone, and Walter's (1985) Zonobiome 2 (Tropical with Summer Rain). Mean annual temperature is 25.2°C, and freezing temperatures are rarely if ever reached; available weather records from the only long‐term station in the Keys (Key West: 1948–2016) show that temperatures never dip below 5°C. Precipitation averages 1178 mm per year, with about three‐quarters falling during the 6‐month May–October period (Ross et al. 1992). Tropical cyclones are a frequent and potent ecological force, as suggested by a mean return interval of 15 years for landfalling hurricanes of Category 3 or higher (Keim et al. 2007).

The Keys are low, limestone islands. In Key Largo, the surface rock is derived from a coral reef deposited parallel to the Florida coast about 140,000 years ago (Lidz 2004). The material is low in mineral content and leaves little behind upon dissolution. Maximum elevation in the study area is 3.3 m above sea level, and TDFs (locally called “hammocks”) occupy all naturally vegetated surfaces above 1 m. As forest develops following fire or human disturbance on these well‐drained surfaces, an organic soil (Folist) can form with a depth of 30 cm or more (Ross et al. 2003). Though limited in volume, such soils provide critical storage of water and nutrients to support tree growth.

Our study area comprised a narrow strip of tropical dry forest, 15 km in length and covering an area of 875 ha. Beginning in about 1880, farming was an important component of the Key Largo economy (Viele 1996; Diamond and Ross 2020). Pineapples, tomatoes, lime, and avocado were the leading crops. Farmers cleared the native hammock and planted in solution holes in the limestone. After about 1935, however, market conditions and transportation issues caused agricultural activities to decline sharply, and the abandoned land returned to forest. Later, extensive areas were cleared for prospective residential development, but most of these projects were never completed. Including several tracts burned in extensive ground fires during the 1970's, the forests of North Key Largo formed a mosaic of stands ranging in age over a century or more when it was reserved from further development in 1982. Today the area is co‐managed by the Crocodile Lake National Wildlife Reserve (US Fish and Wildlife Service) and the Dagny Johnson Key Largo Hammocks Botanical State Park (Florida Parks).

### 2.2 Study Design and Field Methods

In the winter of 1993, researchers from the National Audubon Society sampled tree composition along a chronosequence in the North Key Largo, Florida tropical dry forest (Figure 1). Sampling took place less than 6 months after Hurricane Andrew, a Category 5 storm whose center crossed the Florida mainland 20–30 km to the north. Surface winds ranged from 145 to 200 kph (Categories 1 to 3) in the study area, with highest wind speeds at the northernmost end (Landsea et al. 2004). The size and density of live and hurricane‐killed trees were determined in 23 stands that varied in age from 14 years to more than a century since abandonment following agriculture or other land‐clearing activities. Surface substrate at all sites was a coralline limestone bedrock, and the brackish water table ranged from 1 to 4 m below the surface. Stand age was a good predictor of the composition of the pre‐hurricane forest, which transitioned from a semi‐deciduous early successional assemblage to a mature forest dominated by evergreen species. Hurricane‐induced mortality was concentrated among large trees associated with early successional forests (Ross et al. 2001).

In 2013, following 20 years without a significant hurricane, 19 of the stands were resampled, and 7 additional stands were added. In both surveys, sampling in each stand was concentrated on a nested belt transect, 40–100 m in length. A center line was established, and stem diameter at 1.4 m was measured for each tree 1–5 cm DBH that was rooted within 1 m of the line. Trees 5–25 cm DBH were measured within a plot that extended 2 m from the center line, and all trees > 25 cm DBH were measured if they fell within 5 m of the line.

Several types of environmental data were collected. Soil depth was measured in 2015–17 by probing to bedrock with a 1‐cm diameter rod at 25 evenly spaced locations along the axis of each transect. Surface elevation was determined for 10‐m lengths along each transect from LiDAR‐derived United States Geological Survey 3D Elevation Program (3DEP) Datasets with a vertical accuracy of 10 cm root mean square error (RMSE) and a horizontal resolution of 1 m (United States Geological Survey 2021).

We selected 13 functional traits for analysis (Appendix S1). All were deemed relevant to our hypotheses and had exhibited significant among‐species variation in previous work (Subedi 2017; Subedi, Ross, et al. 2019; Subedi, Hogan, et al. 2019). Methods for measurement generally followed those described in Perez‐Harguindeguy et al. (2013). Data for tree height (HT), diameter at breast height, that is, 1.4 m (DBH), crown area (CA), and the presence of multistemmed structure (MULTI) were collected from all trees ≥ 1 cm DBH rooted within 20 × 20 m plots near 10 of the transects. Other traits were estimated based on sub‐samples of individuals within or near the plots.

Five architectural traits were determined for each species. Means for HT:DBH and CA:DBH were determined within each stand and averaged across all plots where the species was present. The percentage of multistemmed trees (MULTI) was also determined based on species averages across plots. MAXHT was the height of the tallest individual among all measured stems in the 10 plots. Wood density (WD) was determined from three 1–2 cm DBH branch samples collected from 3 to 5 individuals per stand. It was calculated as the ratio of the oven‐dry mass of the wood sample divided by the mass of water displaced by its fresh (green) volume (Chave et al. 2006).

Among the six leaf traits, specific leaf area (SLA; leaf area per unit mass), percent nitrogen and phosphorus (N and P), and δ^13^C were determined from three leaves collected from 3 to 5 individuals of each species present in each plot. Recently expanded sun leaves were sampled, or in cases of understory species, the most illuminated leaves on the plant were sampled (Cornwell and Ackerly 2009).

Estimates of leaf longevity and seasonality were based on a 1991–92 study of leaf demography. Two individuals of 21 common Key Largo trees were selected along a trail near one of the transects; as a group, these species comprised 93.4% of the basal area and 94% of tree density across the stands sampled in 1992. Single meristems from two separate branches were selected for monitoring of leaf production/survival. Beginning in January 1991 and continuing at monthly intervals through January 1992, we counted leaf numbers within two cohorts: marked leaves present at the initiation of the study and leaves added during the next 12 months. To estimate leaf longevity, for each species we plotted the total number of leaves surviving from the 1990 cohort (first counted in January 1990 and not counting new leaves added during subsequent months) by month and estimated the half‐life of leaves in days from a curve (either straight line or quadratic function) fit to the plotted data. Estimated leaf longevity for each species was twice the leaf half‐life. Leaf seasonality was calculated as the coefficient of variation of the total number of leaves counted by month (including new leaves added), from January 1991 to January 1992.

### 2.3 Analytical Methods

We used a principal component analysis (PCA) to produce a detailed ordination of tree species by functional traits. Of the 13 functional traits used in this study, we expected some correlation among traits, especially among traits defined as mathematical functions of other traits (e.g., N, P, and N:P). We expected the PCA to identify such correlations, which we took into account when interpreting results of subsequent ordinations (i.e., NMDS ordinations).

We used a plot of leaf longevity vs. leaf seasonality (leaf longevity and leaf seasonality vectors were approximately orthogonal to one another in the PCA of tree species by functional traits) to group species into three functional groups. We used these two traits to delineate functional groups not only because the trait vectors were orthogonal on the PCA plot, but also because the functional groups were defined by Givnish (2002) by leaf longevity and seasonality, and are related to the leaf economics spectrum (Wright et al. 2004). We analyzed differences in functional traits by functional group using a one‐way MANOVA followed by univariate ANOVAs conducted individually for each functional trait. Following statistically significant (*p* < 0.05) ANOVAs, we used Tukey's post hoc tests to detect differences in functional traits among functional groups. We then quantified changes in mean basal area of the functional groups over the chronosequence of sites.

We used nonmetric multidimensional scaling (NMDS; “metaMDS” function in “Vegan” package in R) to examine relationships among communities and combined it with a vector‐fitting technique to understand the relationships between forest composition and important environmental variables. The ordination was applied to tree species abundances from all 27 sites sampled in 1993 and/or 2013, a total of 71 site‐time combinations. We chose basal area as the measure of abundance, relativized to the maximum value observed for each species (Faith et al. 1987). We used the Bray‐Curtis metric as a measure of dissimilarity among sites. The environmental variables of interest—stand age, soil depth, and elevation—were standardized to mean = 0 and standard deviation = 1 prior to being fit to the ordination. We used the “envfit” function in the “Vegan” package in R to fit the stand age, soil depth, and elevation environmental vectors to each NMDS ordination. For each environmental vector, statistical significance of fit to the ordination was calculated through a permutation test, and goodness of fit was the squared correlation coefficient (*r* ^2^).

A similar ordination was performed on a site‐by‐functional trait data set. The functional trait values were community‐weighted means (CWM) (Violle et al. 2007; Hulshof et al. 2013), that is:

$${\mathrm{CWM}}_{\mathrm{ip}}=\sum \mu_{i}f_{i}$$

where *i* = species, *p* = site and μ_*i*_ and f_*i*_ are the mean trait value and relative abundance (proportion of stand basal area) of the species *i* in site *p*.

Because NMDS is a nonparametric ordination technique designed for nonlinear relationships among variables with no assumption of non‐collinearity among variables (Souza 2025), we expected effects of any correlation among community‐weighted traits on results of the ordinations to be minimal.

To test for convergence in composition and function across the chronosequence, we used the “dispRity” package in R to compare inter‐site dissimilarities (disparities) within three age classes of sites, as sampled in 2013: ≤ 50 (*n* = 6 sites), 51–90 (*n* = 14 sites), and > 90 years since abandonment (*n* = 5 sites). The “dispRity” package measures and compares the volume in multivariate space occupied by sites in each of the age classes (Guillerme 2018). We conducted this analysis twice—once using the 3‐dimensional NMDS ordination of species basal area data for sites and once using a similar ordination of community‐weighted trait data. To identify functional traits driving patterns of inter‐site dissimilarities across the chronosequence, we calculated coefficients of variation (CV) of all community‐weighted traits for each of the three site age classes and compared trends in coefficients of variation among all functional traits across the age classes.

## 3 Results

Twenty‐one tree species > 1 cm dbh were sampled in 23 sites in 1994 and in 25 sites in 2013 (Appendix S2). In the PCA of tree species by functional traits, PCA1 and PCA2 explained a cumulative 49.8% of the variation in species data (randomization test for PCA1 *p* = 0.001, randomization test for PCA2 *p* = 0.023). Species generally separated into two groups: species with high wood density, high leaf longevity, high ratio of crown area to dbh, high proportion of multiple stems, less negative δ13C values, low leaf seasonality, low SLA and low maximum height; and species with low wood density, low leaf longevity, low ratio of crown area to dbh, low proportion of multiple stems, less negative δ^13^C values, high leaf seasonality, high SLA and high maximum height (Appendix S3). Leaf longevity and leaf seasonality vectors were nearly orthogonal to one another (Appendix S3).

Using a plot of species leaf longevity vs. leaf seasonality, we identified three functional groups of tree species: deciduous species, leaf exchangers and evergreen species (Appendix S4). Deciduous species had higher mean leaf seasonality than leaf exchanger or evergreen species (Appendix S5, Appendix S6). Mean leaf longevity for evergreen species, at 701 ± 83 days, was higher than the means for leaf exchanger (*x* ¯ = 368 ± 23 days) and deciduous species (*x* ¯ = 223 ± 18 days; Appendix S5, Appendix S6). The functional groups also differed in a few structural traits. Evergreen species had higher mean wood density than deciduous species; leaf exchanger wood density did not differ from either of the other two functional groups (Appendix S5, Appendix S6). Evergreen species also had a higher mean ratio of crown area to dbh than leaf exchanger species; the mean for deciduous species did not differ from the other functional groups (Appendix S5, Appendix S6). Functional groups showed no differences among the remaining functional traits (Appendix S5).

**FIGURE 2:**
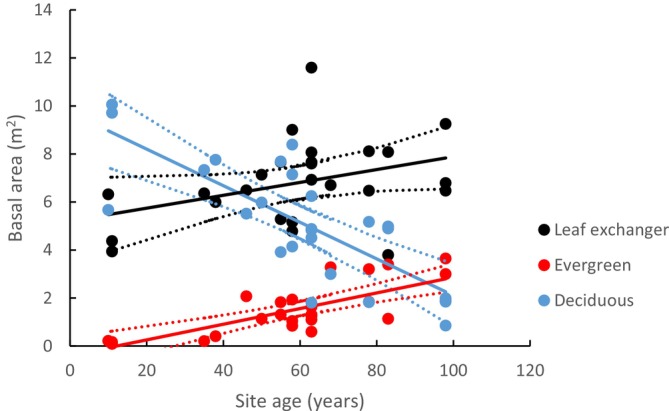
Relative basal areas of species in deciduous, leaf exchanger, and evergreen functional groups measured along a chronosequence of 25 dry deciduous forests in North Key Largo, Florida. Solid and dotted lines represent linear regression lines and 95% confidence bands, respectively.

Sites early in succession (< 30 years old) comprised almost exclusively species in the deciduous and leaf exchanger functional groups, with deciduous species dominating the youngest sites. With increasing age over the chronosequence, mean basal area of deciduous species declined steeply (regression of deciduous basal area on site age: *y* = −0.076*x* + 9.73, R^2^ = 0.58, *p* < 0.0001), mean basal area of leaf exchanger species remained equal (regression of leaf exchanger basal area on site age: *y* = 0.027*x* + 5.21, R^2^ = 0.14, *p* = 0.061), and mean basal area of evergreen species increased slowly from virtually zero in the youngest sites to values close to deciduous species mean values in the oldest sites (regression of evergreen basal area on site age: *y* = 0.033*x*−0.396, *R* ^2^ = 0.57, *p* < 0.0001; Figure 2).

**FIGURE 3:**
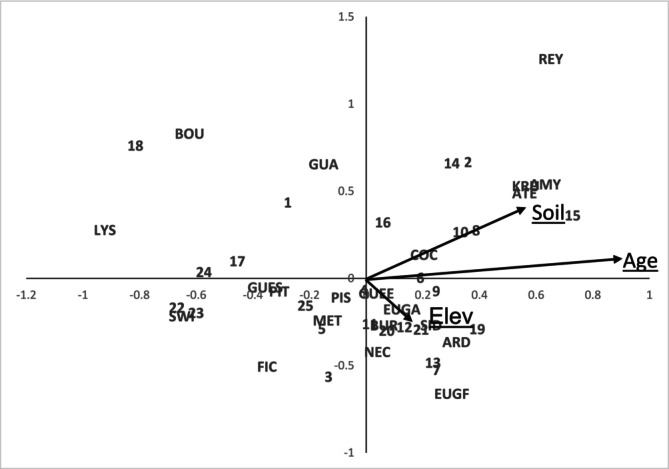
First two axes of NMDS ordination of tree species composition data for 25 dry tropical forests sampled in 2013 in North Key Largo, Florida. Sites are represented by numbers. Species are represented by species codes (first three letters of genus and first three letters of specific epithet; e.g., FICCIT = *Ficus citrifolia* ). Vectors represent strength and direction of regressions of stand age (Age), soil depth (Soil depth) and elevation (Elev) environmental vectors on NMDS1.

**FIGURE 4:**
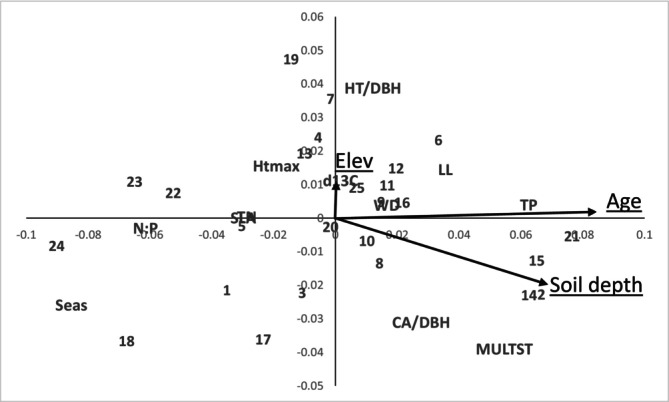
First two axes of NMDS ordination of community‐weighted trait data for 25 dry tropical forests sampled in 2013 in North Key Largo, Florida. Sites are represented by numbers. Traits are leaf seasonality (Seas), leaf nitrogen: phosphorus (N:P), specific leaf area (SLA), maximum height (Htmax), leaf total nitrogen (TN), height: DBH (HT/DBH), δ13C (d13C), wood density (WD), leaf total phosphorus (TP), leaf longevity (LL), canopy area: DBH (CA/DBH), and proportion multiple stems (MULST). Vectors represent strength and direction of regressions of stand age (Age), soil depth (Soil depth), and elevation (Elev) environmental vectors on NMDS1.

Each ordination of sites (ordinations of 1993 and 2013 species and community‐weighted trait data) showed a pronounced change in sites with increasing age along the chronosequence. Regressions of environmental vectors (site age, soil depth and elevation) on the first axis of all ordinations showed a positive relationship with site age and soil depth, but not with elevation (Table 1, Figures 3 and 4). Soil depth appeared positively correlated with site age in each ordination (Figures 3 and 4). In the species composition ordination, most species with low leaf longevity, high leaf seasonality, low wood density, high SLA and high maximum height had negative scores on NMDS1, associated with young site age; while most species with high leaf longevity, low leaf seasonality, high wood density, low SLA and low maximum height had positive scores on NMDS1, associated with older site age. In the ordination on community‐weighted trait data, sites with high positive scores on NMDS1, associated with older site age, had high community‐weighted trait values for leaf longevity, total phosphorus, wood density, proportion of multiple stems, and ratio of crown area to dbh. Site with high negative scores on NMDS1, associated with younger site age, had high community‐weighted trait values for leaf seasonality, SLA, total nitrogen, N:P ratio, and maximum height. When we examined individual sites for directional movement in ordination space along the site age vector over a 19 year time period, movement was positive with respect to species (mean difference in NMDS score from 1994 to 2013 = 0.096 ± 0.035, *t*_20_ = 2.74, one‐tailed *p* = 0.006; Appendix S7), and trended positive with respect to traits (mean difference in NMDS score from 1994 to 2013 = 0.009 + 0.005, *t*_20_ = 1.70, one‐tailed *p* = 0.052; Appendix S8).

**TABLE 1:** Results of NMDS ordinations conducted on species basal areas and community‐weighted traits for 23 dry tropical forest sites sampled in 1994, and 25 sites sampled in 2013 in North Key Largo, Florida.

Variable	Year	Stress	Regression of vectors on NMDS1					
			Site age		Soil depth		Elevation	
			*R* ^2^	*p*	*R* ^2^	*p*	*R* ^2^	*p*
Species	1994	0.131	0.42	0.01	0.74	0.001	0.15	0.266
Species	2013	0.138	0.78	0.001	0.50	0.002	0.15	0.171
Traits	1994	0.042	0.47	0.003	0.49	0.003	0.10	0.412
Traits	2013	0.034	0.72	0.001	0.55	0.001	0.01	0.942

**FIGURE 5:**
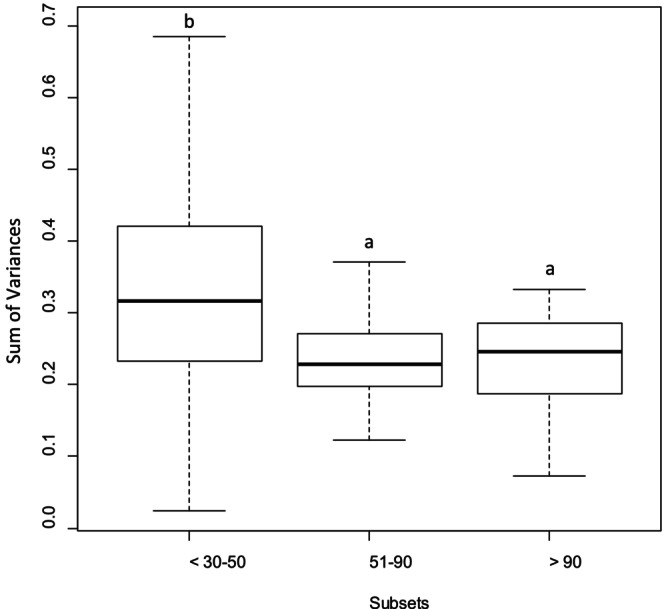
Medians of disparity in species ordination space for 25 dry tropical forests sampled in North Key Largo in 2013, grouped into < 30–50 year (*n* = 6 sites), 51–90 year (*n* = 14 sites), and > 90 year (*n* = 5 sites) age classes. Disparities were measured as the sums of variances of site ordination scores from three NMDS ordination axes. Different letters above bars represent statistically significant differences (*p* < 0.05).

Our analysis of disparity in species ordination space among sites in three age classes showed that median disparity was highest in sites ≤ 50 years old and lower in sites > 50 years old (Figure 5).

**FIGURE 6:**
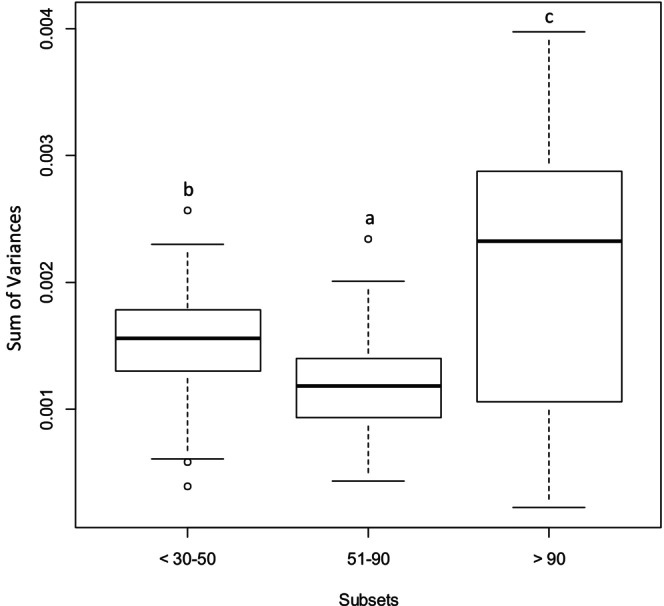
Medians of disparity in trait ordination space for 25 dry tropical forests sampled in North Key Largo in 2013, grouped into < 30–50 year (*n* = 6 sites), 51–90 year (*n* = 14 sites), and > 90 year (*n* = 5 sites) age classes. Disparities were measured as the sums of variances of site ordination scores from three NMDS ordination axes. Different letters above bars represent statistically significant differences (*p* < 0.05).

Analysis of disparity in trait ordination space among sites in the same age classes showed that median disparity decreased from the ≤ 50 year to 51–90 year age class but then increased to the highest median disparity in the old growth sites > 90 years old (Figure 6).

The general trend in CVs of most of the community‐weighted traits across the age classes followed the trend in median disparities, with relatively high CV values in the ≤ 50 year age class, then decreased values in the 51–90 year age class, followed by high values in the > 90 year age class. Three of the five functional traits with highest CV values in the oldest age class were related to tree structure (e.g., maximum height, height/dbh, and proportion of multiple stems). CV values for proportion of multiple stems had the most pronounced pattern, with the highest value of all the functional traits in the youngest sites, and a CV value in the oldest sites 50% higher than the next highest CV value. The only functional trait with a markedly different pattern of CV values was leaf seasonality, with lower CV values in the youngest and oldest age classes, and the highest CV value in the 51–90 year age class (Appendix S9).

## 4 Discussion

Similar to previous studies in Florida Keys TDFs (Ross et al. 2001; Subedi, Ross, et al. 2019), the chronosequence of sites in this study demonstrated a clear pattern of resource‐acquisitive species having highest abundance early in succession, while resource‐conserving species had highest abundance late in succession (Figures 3 and 4). This study, however, is one of the first in this ecosystem to corroborate the successional pattern shown by a chronosequence with temporal changes in individual sites. As predicted, species functional groups delineated by leaf seasonality and leaf longevity showed a growth‐survival demographic tradeoff between deciduous species early in succession and evergreen species late in succession. Unexpectedly, however, we identified a third functional group, leaf‐exchangers, which attained relatively high abundance early in succession and reached dominance by late succession. Additionally, while species composition demonstrated convergence over the course of succession as expected, functional trait composition surprisingly demonstrated a lack of convergence (Figures 5 and 6). We suggest that this divergence of functional traits late in succession is due to leaf‐exchangers and evergreen species coexisting with relatively high abundances and contrasting functional traits. Correlation of environmental variables and small sample size of sites late in succession, however, necessitates cautious interpretation of these results.

### 4.1 Species Functional Traits

Through the PCA of species by functional traits, we differentiated between species with resource‐acquisitive traits‐high leaf seasonality and low leaf longevity, high SLA, more negative δ^13^C values, low wood density, and high maximum height—and species with resource‐conservative traits‐low leaf seasonality and high leaf longevity, low SLA, less negative δ^13^C values, high wood density and low maximum height (Appendix S3, Bazzaz and Pickett 1980, Swenson et al. 2012, Subedi, Ross, et al. 2019). Focusing on functional traits and species associated with strategies along the leaf economics spectrum, however, yields a more nuanced representation of successional dynamics. In the PCA of species by functional traits, vectors for leaf seasonality and leaf longevity, primary traits underpinning the leaf economics spectrum, were approximately orthogonal to each other (Appendix S3). This orthogonality indicates that these traits respond to different environmental stimuli or gradients during succession. We suggest that leaf seasonality and leaf longevity represent and are responses to successional soil moisture availability and light availability gradients, respectively.

We used species‐specific values for leaf longevity and leaf seasonality to define deciduous, leaf‐exchanger, and evergreen species groups along the leaf economics spectrum. Deciduous species, with high leaf seasonality, had the highest abundance in early successional sites. Open canopies and shallow to no soils in these sites almost certainly result in low soil moisture availability, particularly during the dry season. Deciduous species' drought avoidance mechanism of losing leaves during the dry season enables them to minimize energetic losses during the unfavorable season while maintaining high photosynthetic and growth rates during the favorable wet season (Givnish 2002). *Lysiloma bahamensis*, a relatively well‐studied, distinctive member of this functional group, recruits seedlings primarily from a dormant seed bank following high‐intensity disturbances such as land clearing, fires, or major hurricane damage (Pascarella 1997), with populations having the highest abundances early in succession.

Evergreen species, with high leaf longevities, had highest abundance in late successional sites with low light availability. Holding leaves for longer periods of time enables these species to survive in low‐light conditions through maintaining relatively low photosynthetic and growth rates throughout the year and reducing leaf construction and nutrient replacement costs through amortization (Givnish 2002). Leaf‐exchangers generally had functional traits intermediate between deciduous and evergreen species, holding leaves for approximately a year like deciduous species, but lacking a leafless period like evergreen species. Short‐lived leaves with SLA values intermediate between deciduous and evergreen species enabled leaf‐exchangers to grow relatively rapidly early in succession, while maintaining leaves year‐round enabled persistence in stands through late succession. Little is known about regeneration characteristics or population demographies of evergreen or leaf exchanger species in Florida Keys TDFs.

### 4.2 Successional Change in Species Composition, Functional Traits

A pronounced change in both species abundances and community‐weighted traits occurred in sites along the successional chronosequence, as shown by the strong association of the first axis of all site ordinations with site age. The first axis of all site ordinations also was strongly associated with soil depth (Figures 3 and 4); thus, increasing soil depth during succession might be expected to increase resources (e.g., water, nutrients) available to species. The direction of change along the site age axis in species and their associated traits, however, suggests that instead, resources became increasingly limited as succession progressed. Species and community‐weighted traits associated with resource acquisition and fast growth had high prevalence in early successional sites, whereas species and community‐weighted traits associated with resource conservation and slow growth had high prevalence in late successional sites. Although soil depth increased with increasing site age, soil in the oldest sites comprised primarily organic matter no more than 28 cm deep over porous limestone rock with a brackish water table sometimes overlain by a shallow freshwater lens. In the context of this belowground environment, low light availability due to canopy closure is likely the primary limiting resource for tree species late in succession. Thus, the site age axis represents a successional gradient from low soil moisture and nutrients, but high light availability early in succession; to higher soil moisture and nutrients, but low light availability late in succession.

This pattern of change in functional traits along a chronosequence agrees with results of an earlier successional study conducted in nearby sites in the Florida Keys (Subedi, Ross, et al. 2019) and has occurred in successional studies of some subtropical and tropical wet forests (Swenson et al. 2012; Muscarella et al. 2016). Patterns of functional trait change during succession in other TDFs, however, have been either equivocal (Lebrija‐Trejos et al. 2010), or opposite to the pattern shown here (Buzzard et al. 2016).

Individual sites showed similar directional change in species abundances and community‐weighted traits over the 20 years of this study, as we predicted. Although sites were recovering from Hurricane Andrew at the beginning of the study, disturbance from the hurricane did not appear to alter successional trajectories of sites. Unlike forest communities on the Florida mainland north of the study area that sustained much more intense damage from the hurricane (Horvitz et al. 1995), our sites experienced no significant invasions from exotic vine species, and forest canopies recovered rapidly.

### 4.3 Evidence for Convergence in Species, Functional Traits

Both the pronounced directional change in species shown in individual sites, and the decreased disparity shown among sites > 50 years old in species multivariate space are evidence for deterministic community assembly and convergence among sites in species as succession progresses (Figure 5). With respect to disparity among sites in community‐weighted trait multivariate space, instead of indicating convergence among sites in traits, the data showed the highest variation in traits among the oldest sites (Figure 6). Both patterns of change in trait multivariate space within individual sites over time, and patterns of disparity in trait multivariate space among sites over the successional chronosequence showed relatively high variability related to functional traits, especially early and late in succession.

Disparity for each age class of sites is defined as the sum of variances on each of the three NMDS multivariate dimensions; thus, disparities in both species and community‐weighted trait multivariate space can be thought of as 3‐dimensional volumes in species‐ or trait‐space (Guillerme 2018). Moreover, disparity values in species‐ and trait‐space, because they measure variation among sites, are analogous to species or functional beta diversity.

For sites in the oldest age class (> 90 years old), disparity in species‐space was low, while disparity in trait‐space was high, similar to sites having high functional beta diversity compared to species beta diversity. Most studies comparing species and functional beta diversities have shown tropical communities to have low functional beta diversity relative to species beta diversity, likely a result of habitat filtering due to the strong influence of environmental conditions leading to functional redundancy among species (Swenson et al. 2011; Fu et al. 2019; Villa et al. 2021). The present study is one of just a few studies suggesting that functional beta diversity late in succession is high relative to species beta diversity. Communities with higher‐than‐expected functional beta diversity compared to species beta diversity may be due to species in the communities being more functionally different than expected given the species pool, likely due to competition during community assembly resulting in functionally divergent species co‐occurring (Villa et al. 2021). Alternatively, functional differences in species among sites may be due to environmental heterogeneity among sites resulting from disturbances, rather than simply due to dispersal limitation (Swenson et al. 2011; Muscarella et al. 2016).

Although relatively frequent hurricanes and smaller‐scale disturbances causing canopy gaps occur in old‐growth Florida Keys TDFs and might increase environmental heterogeneity, it is unlikely that this mechanism is a primary driver of high functional disparity in the conventional manner—by providing recruitment opportunities for early successional species. Previous studies have shown that, conversely, canopy gaps and disturbances by hurricanes promote recruitment and resprouting by relatively late‐successional species in these forests (Ross et al. 2001, Diamond and Ross 2016).

Results from this study suggest that high functional disparity in late successional sites results from a combination of species replacement driven by life‐history trade‐offs (van Breugel et al. 2024) and interspecific competition late in succession. As has been shown in earlier studies in this ecosystem and in additional TDFs and tropical wet forests (Swenson et al. 2012; Muscarella et al. 2016; Subedi, Ross, et al. 2019), this study demonstrated species replacement driven by the life‐history trade‐off between resource capture and rapid growth (i.e., “fast” species) and resource conservation and high survival (i.e., “slow” species; Ruger et al. 2023, van Breugel et al. 2024). We maintain that “fast” and “slow” species in this system are deciduous and evergreen species, respectively. In addition, however, this species replacement included leaf‐exchanger species that grew rapidly to attain high abundance early in succession, then survived through late succession as a significant component of the forest canopy.

We describe the species replacement sequence in this study through the dynamics of deciduous species associated with resource acquisition, evergreen species associated with resource conservation, and leaf‐exchangers, and relate these dynamics to changes in functional trait disparities among sites at different stages during succession. Sites ≤ 50 years old (the youngest sites) had high functional trait disparity, although disparity was not as high as that shown among the oldest sites. This relatively high disparity in trait‐space was corroborated by high variability in temporal directional change in trait multivariate space in individual sites and indicates that community assembly early in succession is more stochastic than deterministic, likely due to species highly variable in functional traits establishing early in succession. Deciduous species and leaf‐exchangers were well‐represented with relatively high basal areas in these sites. Sites in the 51–90 year age class had low disparities in both species‐ and trait‐space. By this stage in succession, basal areas of deciduous species had declined precipitously, leaving sites dominated by leaf‐exchangers. In the oldest sites, with the highest functional trait disparity, leaf‐exchangers remained with relatively high basal areas, and were joined by evergreen species that had increased in abundance by this stage in succession. Although the addition of evergreen species to the community increased the disparity in species‐space slightly (but not statistically significantly), this addition substantially increased disparity in trait‐space, likely because the functional traits within and between the two dominant demographic groups (evergreen species and leaf‐exchangers) differed more than at any other stage in succession. Patterns in CV values of individual functional traits across successional age classes largely corroborated patterns in functional trait disparity. Most functional traits had higher CV values early and late in succession, with canopy area/DBH (which differed between evergreen and leaf exchanger species) having the highest CV value in the oldest sites (Appendix S9).

Although these dynamics among functional groups possibly were drivers of high functional trait disparity late in succession, we must emphasize that the results of the disparity analysis in functional trait space must be interpreted with caution due to two caveats: correlation between the stand age and soil depth environmental vectors, and small sample size of stands late in succession. The NMDS analyses showed that stand age and soil depth were strongly correlated, which makes it difficult to determine which of the two environmental variables is a primary cause of high functional trait disparity late in succession. Although our interpretation assumes that stand age is the major environmental factor associated with these results, an alternative explanation might be that soil depth is the primary factor, and that deep soils of sites late in succession support more functionally distinct communities than sites on shallower soils earlier in succession. Also complicating interpretation of these results is the small number of sites greater than 90‐years‐old; five sites were in this age class. High functional trait disparity among sites in this age class could be due primarily to large variance due to small sample size; For Example, disproportionate effects on the results from one or more “outlier” sites.

Results of this study are progress toward increased understanding of community dynamics of an imminently threatened ecosystem. TDFs are among the most threatened ecosystems worldwide (Janzen 1988; Stan et al. 2024) and generally function differently from and are less well understood than wet tropical forests (Dexter et al. 2018). Florida Keys TDFs, moreover, are members of a narrowly‐distributed variant of coastal TDFs that occurs on limestone substrates in the northeastern Caribbean region—located in the northern Bahamas, southwestern Puerto Rico, and the Florida Keys. These ecosystems with a distinctive combination of limestone substrate, low‐elevation coastal locations, and ensuing vulnerability to tropical storms and sea level rise (Freeman et al. 2024) might be expected to respond to disturbances differently than other TDFs, and this study adds to evidence from past studies (Ross et al. 2001; Carrington et al. 2015; Subedi, Ross, et al. 2019) that Florida Keys TDFs do, in fact, respond differently. This study showed that although species composition converged during succession in a deterministic fashion, functional traits did not and instead were most variable in the oldest sites. This surprising result seems to reflect co‐occurrence of canopy species from two demographic groups (i.e., leaf‐exchangers and evergreen species) with quite different functional traits, along with interspecific competition for limited resources. Additional study of interspecific competition and recruitment resulting from small‐scale disturbances in old‐growth Florida Keys TDFs is needed to better understand community dynamics late in succession.

## Author Contributions

**Mary E. Carrington:** conceptualization (equal), data curation (equal), formal analysis (lead), methodology (supporting), project administration (supporting), validation (equal), writing – original draft (lead), writing – review and editing (equal). **Michael S. Ross:** conceptualization (equal), formal analysis (supporting), funding acquisition (lead), investigation (lead), methodology (lead), project administration (equal), supervision (equal), validation (equal), writing – original draft (supporting), writing – review and editing (equal). **Suresh C. Subedi:** conceptualization (equal), data curation (equal), formal analysis (supporting), funding acquisition (equal), investigation (equal), methodology (equal), project administration (equal), supervision (equal), validation (equal), writing – original draft (supporting), writing – review and editing (equal).

## Conflicts of Interest

The authors declare no conflicts of interest.

## Supporting information


**Appendix S1:** List of measured traits, abbreviations, and units (Table S1).
**Table S1:** List of traits measured traits, abbreviations (parenthesis) and units.

**Appendix S2:** Names, codes, and functional groups of tree species sampled in 23 dry tropical forest sites in 1994, and 25 sites in 2013 in North Key Largo, Florida (Table S2).
**Table S2:** Names, codes, and functional groups of tree species sampled in 23 dry tropical forest sites in 1994, and 25 sites in 2013 in North Key Largo, Florida.

**Appendix S3:** PCA of 21 tree species present in North Key Largo dry tropical forests (Figure S1).
**Figure S1:** PCA of 21 tree species present in North Key Largo dry tropical forests. Vectors represent loadings of functional traits on the two axes. Traits are leaf seasonality (Seas), leaf nitrogen: phosphorus (N:P), specific leaf area (SLA), maximum height (Htmax), leaf total nitrogen (TN), height:dbh (HT/DBH), δ13C (d13C), wood density (WD), leaf total phosphorus (TP), leaf longevity (LL), canopy area:dbh (Cr/DBH), and proportion multiple stems (mult).

**Appendix S4:** Scatter plot of leaf longevity vs. coefficient of variation of number of leaves across months for 21 dry tropical forest tree species in North Key Largo, Florida (Figure S2).
**Figure S2:** Scatter plot of leaf longevity vs. coefficient of variation of number of leaves across months for 21 dry tropical forest tree species in North Key Largo, Florida. Species are grouped into deciduous, leaf exchanger and evergreen functional groups.

**Appendix S5:** MANOVA and univariate test results for 12 functional traits of dry tropical forest tree species, comparing means among three functional groups of species (evergreen, leaf exchanger, and deciduous) (Table S3).
**Table S3:** MANOVA and univariate test results for 12 functional traits of dry tropical forest tree species, comparing means among three functional groups of species (evergreen, leaf exchanger, and deciduous; fixed treatment effect = Functional Group). *p*‐values of statistically significant (*p* < 0.05, *p* < 0.01, *p* < 0.001) univariate tests are in bold font. For the multivariate test statistic (Pillai's trace), dfn is based on the of factor levels (3) and number of response variables (12), while dfd is the residual degrees of freedom of the model.

**Appendix S6:** Means of leaf seasonality (a), leaf longevity (b), wood density (c), ratio of crown area to dbh (d), and SLA (e) for dry tropical tree species in deciduous, leaf exchanger and evergreen functional groups (Figure S3).
**Figure S3:** Means of leaf seasonality (a), leaf longevity (b), wood density (c), ratio of crown area to dbh (d), and SLA (e) for dry tropical tree species in deciduous, leaf exchanger and evergreen functional groups. N = 7 species in each functional group. Error bars are standard errors. Different letters above bars represent statistically significant differences (Tukey's post hoc tests, *p* < 0.05).

**Appendix S7:** First two axes of NMDS ordination of tree species composition data for 21 dry tropical forests in ≤ 50 year, 51–90 year, and > 90 year age classes, sampled in 1993 and 2013 in North Key Largo, Florida (Figure S4).
**Figure S4:** First two axes of NMDS ordination of tree species composition data for 21 dry tropical forests in ≤ 50 year, 51–90 year, and > 90 year age classes, sampled in 1993 and 2013 in North Key Largo, Florida. NMDS axes are rotated to the Age environmental vector fit to the ordination. Arrows represent differences in individual site coordinates from 1993 to 2013. Bold arrows represent mean change among sites in each age class.

**Appendix S8:** First two axes of NMDS ordination of community‐weighted trait data for 21 dry tropical forests in ≤ 50 year, 51–90 year, and > 90 year age classes, sampled in 1993 and 2013 in North Key Largo, Florida (Figure S5).
**Figure S5:** First two axes of NMDS ordination of community‐weighted trait data for 21 dry tropical forests in ≤ 50 year, 51–90 year, and > 90 year age classes, sampled in 1993 and 2013 in North Key Largo, Florida. NMDS axes are rotated to the Age environmental vector fit to the ordination. Arrows represent differences in individual site coordinates from 1993 to 2013. Bold arrows represent mean change among sites in each age class.

**Appendix S9:** Coefficients of variation for 12 community‐weighted functional traits from 25 dry tropical forests sampled in North Key Largo in 2013, grouped into < 30–50 year, 51–90 year, and > 90 year age classes (Figure S6).
**Figure S6:** Coefficients of variation for 12 community‐weighted functional traits from 25 dry tropical forests sampled in North Key Largo in 2013, grouped into < 30–50 year, 51–90 year, and > 90 year age classes.

## Data Availability

Data and data analysis files are available in a Dryad dataset: DOI https://doi.org/10.5061/dryad.c2fqz61rt.
