# Supplementary material for: Succession in a Tropical Dry Forest: A Test of the Chronosequence and Inference of Community Assembly Dynamics
Source: Ecol Evol. 2026 Jun 23;16(6):e73895. doi: 10.1002/ece3.73895 (PMC13288376; doi:10.1002/ece3.73895)
Supplement: Supplementary file 1 — Appendix S1: List of measured traits, abbreviations, and units (Table S1). Table S1: List of traits measured traits, abbreviations (parenthesis) and units. [file ECE3-16-e73895-s003.docx]

Supplementary Table 1. List of traits measured traits, abbreviations (parenthesis) and units.

| Trait | Unit |
| --- | --- |
| Maximum height (MaxHT) | m |
| Specific leaf area (SLA) | cm^2^g^-1^ |
| Wood density (WD) | gcm^-3^ |
| Leaf total phosphorus (TP) | μgg^-1^ |
| Leaf total nitrogen (TN) | % |
| Leaf stable carbon isotope ratio (δ^13^C) | ‰ |
| Leaf N:P (N:P) | ratio |
| Crown area:Diameter at breast height (CA:DBH) | cm |
| Height:Diameter at breast height (HT:DBH) | ratio |
| Height:Crown length (HT:CL) | ratio |
| Frequency of multiple stems (MULTI) | % |
| Leaf longevity (LL) | Number of days |
| Leaf seasonality (SEAS) | Number of days |
