# Supplementary material for: Succession in a Tropical Dry Forest: A Test of the Chronosequence and Inference of Community Assembly Dynamics
Source: Ecol Evol. 2026 Jun 23;16(6):e73895. doi: 10.1002/ece3.73895 (PMC13288376; doi:10.1002/ece3.73895)
Supplement: Supplementary file 2 — Appendix S2: Names, codes, and functional groups of tree species sampled in 23 dry tropical forest sites in 1994, and 25 sites in 2013 in North Key Largo, Florida (Table S2). Table S2: Names, codes, and functional groups of tree species sampled in 23 dry tropical forest sites in 1994, and 25 sites in 2013 in North Key Largo, Florida. [file ECE3-16-e73895-s008.docx]

Supplementary Table 2. Names, codes, and functional groups of tree species sampled in 23 dry tropical forest sites in 1994, and 25 sites in 2013 in North Key Largo, Florida.

| **Species Name** | **Code** | **Functional Group** |
| --- | --- | --- |
| *Amyris elemifera* | AMY | Evergreen |
| *Ardisia escallonioides* | ARD | Evergreen |
| *Ateramnus lucida* | ATE | Evergreen |
| *Bourreria ovata* | BOU | Leaf exchanger |
| *Bursera simaruba* | BUR | Deciduous |
| *Coccoloba diversifolia* | COC | Leaf exchanger |
| *Eugenia axillaris* | EUGA | Evergreen |
| *Eugenia foetida* | EUGF | Leaf exchanger |
| *Ficus citrifolia* | FIC | Deciduous |
| *Guapira discolor* | GUA | Leaf exchanger |
| *Guettarda elliptica* | GUEE | Deciduous |
| *Guettarda scabra* | GUES | Deciduous |
| *Krugiodendron ferreum* | KRU | Evergreen |
| *Lysiloma bahamensis* | LYS | Deciduous |
| *Metopium toxiferum* | MET | Leaf exchanger |
| *Nectandra coriacea* | NEC | Evergreen |
| *Piscidia piscipula* | PIS | Deciduous |
| *Pithecellobium guadeloupense* | PIT | Leaf exchanger |
| *Reynosia septentrionalis* | REY | Evergreen |
| *Sideroxylon salicifolium* | SID | Leaf exchanger |
| *Swietenia mahagoni* | SWI | Deciduous |
