# Supplementary material for: Succession in a Tropical Dry Forest: A Test of the Chronosequence and Inference of Community Assembly Dynamics
Source: Ecol Evol. 2026 Jun 23;16(6):e73895. doi: 10.1002/ece3.73895 (PMC13288376; doi:10.1002/ece3.73895)
Supplement: Supplementary file 3 — Appendix S3: PCA of 21 tree species present in North Key Largo dry tropical forests (Figure S1). Figure S1: PCA of 21 tree species present in North Key Largo dry tropical forests. Vectors represent loadings of functional traits on the two axes. Traits are leaf seasonality (Seas), leaf nitrogen: phosphorus (N:P), specific leaf area (SLA), maximum height (Htmax), leaf total nitrogen (TN), height:dbh (HT/DBH), δ13C (d13C), wood density (WD), leaf total phosphorus (TP), leaf longevity (LL), canopy area:dbh (Cr/DBH), and proportion multiple stems (mult). [file ECE3-16-e73895-s002.docx]

Supplementary Figure 1. PCA of 21 tree species present in North Key Largo dry tropical forests. Vectors represent loadings of functional traits on the two axes. Traits are leaf seasonality (Seas), leaf nitrogen:phosphorus (N:P), specific leaf area (SLA), maximum height (Htmax), leaf total nitrogen (TN), height:dbh (HT/DBH), δ13C (d13C), wood density (WD), leaf total phosphorus (TP), leaf longevity (LL), canopy area:dbh (Cr/DBH), and proportion multiple stems (mult).
