# Supplementary material for: Succession in a Tropical Dry Forest: A Test of the Chronosequence and Inference of Community Assembly Dynamics
Source: Ecol Evol. 2026 Jun 23;16(6):e73895. doi: 10.1002/ece3.73895 (PMC13288376; doi:10.1002/ece3.73895)
Supplement: Supplementary file 4 — Appendix S4: Scatter plot of leaf longevity vs. coefficient of variation of number of leaves across months for 21 dry tropical forest tree species in North Key Largo, Florida (Figure S2). Figure S2: Scatter plot of leaf longevity vs. coefficient of variation of number of leaves across months for 21 dry tropical forest tree species in North Key Largo, Florida. Species are grouped into deciduous, leaf exchanger and evergreen functional groups. [file ECE3-16-e73895-s009.docx]

*Leaf exchanger*

*Evergreen*

*Deciduous*

Supplementary Figure 2. Scatter plot of leaf longevity vs. coefficient of variation of number of leaves across months for 21 dry tropical forest tree species in North Key Largo, Florida. Species are grouped into deciduous, leaf exchanger and evergreen functional groups.
