# Supplementary material for: Succession in a Tropical Dry Forest: A Test of the Chronosequence and Inference of Community Assembly Dynamics
Source: Ecol Evol. 2026 Jun 23;16(6):e73895. doi: 10.1002/ece3.73895 (PMC13288376; doi:10.1002/ece3.73895)
Supplement: Supplementary file 5 — Appendix S5: MANOVA and univariate test results for 12 functional traits of dry tropical forest tree species, comparing means among three functional groups of species (evergreen, leaf exchanger, and deciduous) (Table S3). Table S3: MANOVA and univariate test results for 12 functional traits of dry tropical forest tree species, comparing means among three functional groups of species (evergreen, leaf exchanger, and deciduous; fixed treatment effect = Functional Group). p‐values of statistically significant (p < 0.05, p < 0.01, p < 0.001) univariate tests are in bold font. For the multivariate test statistic (Pillai's trace), dfn is based on the of factor levels (3) and number of response variables (12), while dfd is the residual degrees of freedom of the model. [file ECE3-16-e73895-s001.docx]

Supplementary Table 3. MANOVA and univariate test results for 12 functional traits of dry tropical forest tree species, comparing means among three functional groups of species (evergreen, leaf exchanger, and deciduous; fixed treatment effect = Functional Group). *P*-values of statistically significant (*p* < 0.05, *p* < 0.01*, p* < 0.001) univariate tests are in bold font. For the multivariate test statistic (Pillai’s trace), dfn is based on the of factor levels (3) and number of response variables (12), while dfd is the residual degrees of freedom of the model.

| 1. **MANOVA** | | | | | | |
| --- | --- | --- | --- | --- | --- | --- |
| Model Factor | df | Pillai’s Trace | dfn | dfd | *F* | *p* |
| Functional Group | 2 | 1.79 | 24 | 16 | 5.75 | 0.0004 |
| 1. **Univariate Tests** | | | | | | |
| Response Variable | | | dfn | dfd | *F* | *p* |
| SLA | | | 2 | 18 | 2.68 | 0.096 |
| WD | | | 2 | 18 | 8.60 | **0.002** |
| MULTI | | | 2 | 18 | 0.69 | 0.513 |
| δ^13^C | | | 2 | 18 | 2.41 | 0.118 |
| TP | | | 2 | 18 | 1.85 | 0.185 |
| TN | | | 2 | 18 | 0.11 | 0.898 |
| N:P | | | 2 | 18 | 0.91 | 0.421 |
| HT:DBH | | | 2 | 18 | 0.22 | 0.807 |
| CA:DBH | | | 2 | 18 | 4.21 | **0.032** |
| MaxHT | | | 2 | 18 | 2.19 | 0.141 |
| LL | | | 2 | 18 | 23.3 | **<0.001** |
| SEAS | | | 2 | 18 | 29.5 | **<0.001** |
