# Supplementary material for: Succession in a Tropical Dry Forest: A Test of the Chronosequence and Inference of Community Assembly Dynamics
Source: Ecol Evol. 2026 Jun 23;16(6):e73895. doi: 10.1002/ece3.73895 (PMC13288376; doi:10.1002/ece3.73895)
Supplement: Supplementary file 6 — Appendix S6: Means of leaf seasonality (a), leaf longevity (b), wood density (c), ratio of crown area to dbh (d), and SLA (e) for dry tropical tree species in deciduous, leaf exchanger and evergreen functional groups (Figure S3). Figure S3: Means of leaf seasonality (a), leaf longevity (b), wood density (c), ratio of crown area to dbh (d), and SLA (e) for dry tropical tree species in deciduous, leaf exchanger and evergreen functional groups. N = 7 species in each functional group. Error bars are standard errors. Different letters above bars represent statistically significant differences (Tukey's post hoc tests, p < 0.05). [file ECE3-16-e73895-s007.docx]

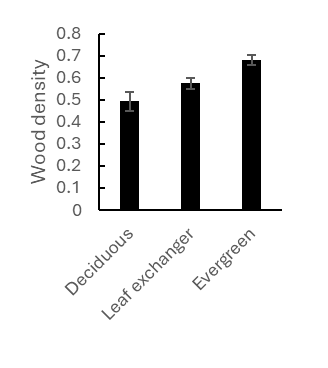


a

ab

b


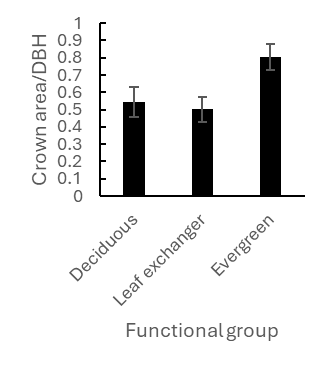


a

b

ab


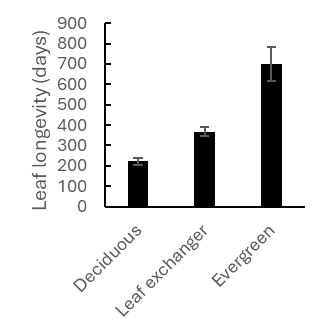


a

a

b


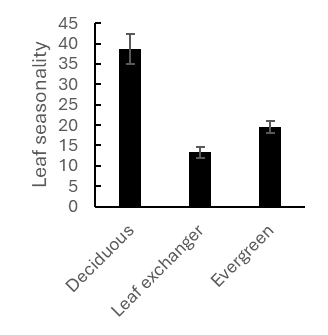


a

a

b

**a.**

**b.**

**c.**

**d.**


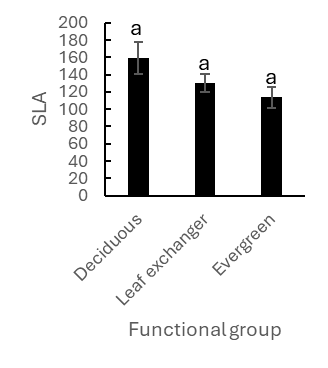


Supplementary Figure 3. Means of leaf seasonality (a.), leaf longevity (b.), wood density (c.), ratio of crown area to dbh (d.), and SLA (e.) for dry tropical tree species in deciduous, leaf exchanger and evergreen functional groups. *N*= 7 species in each functional group. Error bars are standard errors. Different letters above bars represent statistically significant differences (Tukey’s *post-hoc* tests, *p* < 0.05).
