# Supplementary material for: Succession in a Tropical Dry Forest: A Test of the Chronosequence and Inference of Community Assembly Dynamics
Source: Ecol Evol. 2026 Jun 23;16(6):e73895. doi: 10.1002/ece3.73895 (PMC13288376; doi:10.1002/ece3.73895)
Supplement: Supplementary file 8 — Appendix S8: First two axes of NMDS ordination of community‐weighted trait data for 21 dry tropical forests in ≤ 50 year, 51–90 year, and > 90 year age classes, sampled in 1993 and 2013 in North Key Largo, Florida (Figure S5). Figure S5: First two axes of NMDS ordination of community‐weighted trait data for 21 dry tropical forests in ≤ 50 year, 51–90 year, and > 90 year age classes, sampled in 1993 and 2013 in North Key Largo, Florida. NMDS axes are rotated to the Age environmental vector fit to the ordination. Arrows represent differences in individual site coordinates from 1993 to 2013. Bold arrows represent mean change among sites in each age class. [file ECE3-16-e73895-s005.docx]

Supplementary Figure 5. First two axes of NMDS ordination of community-weighted trait data for 21 dry tropical forests in < 50 year, 51 -90 year, and > 90 year age classes, sampled in 1993 and 2013 in North Key Largo, Florida. NMDS axes are rotated to the Age environmental vector fit to the ordination. Arrows represent differences in individual site coordinates from 1993 to 2013. Bold arrows represent mean change among sites in each age class.
