# Supplementary material for: Succession in a Tropical Dry Forest: A Test of the Chronosequence and Inference of Community Assembly Dynamics
Source: Ecol Evol. 2026 Jun 23;16(6):e73895. doi: 10.1002/ece3.73895 (PMC13288376; doi:10.1002/ece3.73895)
Supplement: Supplementary file 9 — Appendix S9: Coefficients of variation for 12 community‐weighted functional traits from 25 dry tropical forests sampled in North Key Largo in 2013, grouped into < 30–50 year, 51–90 year, and > 90 year age classes (Figure S6). Figure S6: Coefficients of variation for 12 community‐weighted functional traits from 25 dry tropical forests sampled in North Key Largo in 2013, grouped into < 30–50 year, 51–90 year, and > 90 year age classes. [file ECE3-16-e73895-s004.docx]

Supplementary Figure 6. Coefficients of variation for 12 community-weighted functional traits from 25 dry tropical forests sampled in North Key Largo in 2013, grouped into <30 – 50 year, 51 – 90 year, and > 90 year age classes.
